# A genome-wide shRNA screen uncovers a novel potential ligand for NK cell activating receptors

**DOI:** 10.3389/fimmu.2025.1537876

**Published:** 2025-06-18

**Authors:** Paolo Romania, Loredana Cifaldi, Paula Gragera, Valerio D’Alicandro, Matteo Caforio, Valentina Folgiero, Valeria Lucarini, Ombretta Melaiu, Roberto Bei, Franco Locatelli, Doriana Fruci

**Affiliations:** ^1^ Bambino Gesù Children’s Hospital, Istituto di Ricovero e Cura a Carattere Scientifico (IRCCS), Rome, Italy; ^2^ Department of Clinical Sciences and Translational Medicine, University of Rome “Tor Vergata”, Rome, Italy; ^3^ Department of Life Sciences, Catholic University of the Sacred Heart, Rome, Italy

**Keywords:** NK cell, activating receptors, ligands, genome-wide screening, cancer immunotherapy, PLAC1, prognostic value

## Abstract

**Introduction:**

Natural Killer (NK) cells play a key role in both innate and adaptive immune responses against viruses and tumor cells. Their function relies on the dynamic balance between activating and inhibitory signals, which are mediated by receptors that bind ligands expressed on target cells. While much is known about the function and expression patterns of NK cell activating receptors (NKARs), many of their ligands remain unidentified.

**Methods:**

K562 cells were transduced with a shRNA library targeting 15,000 genes and co-cultured with NK cells from healthy donors. Surviving clones were tested in cytotoxicity and degranulation assays. PLAC1 was cloned from JEG3 cells in a lentiviral vector and transfected in K562 cells. PLAC1-related gene expression and survival data were obtained from the TCGA database and analyzed using R. PLAC1 and DSG2 expression in healthy tissues and NK cells was obtained from the HPA database and a GEO dataset.

**Results:**

We identified ten candidate genes whose downregulation in K562 cells decreased NK cell-mediated cytotoxicity to levels comparable to silencing the MICA gene. The most promising candidates were functionally validated through single-target gene silencing and overexpression. Among them, the placenta-specific 1 (*PLAC1*) gene stood out, as its inhibition conferred the greatest protection to target cells from NK cell lysis, while overexpression of PLAC1 significantly increased NK cell degranulation. Importantly, PLAC1 was found to interact with NKAR fusion proteins, including NKG2D, DNAM1 NKp44 and NKp30, suggesting its potential involvement in NK cell function. PLAC1 is typically silent in normal tissues, with the exception of placental trophoblasts and testicular germ cells, but is markedly overexpressed in a wide range of tumors. Notably, its prognostic significance appears to be tumor-type specific, associating with either favorable or poor outcomes depending on the cancer context.

**Discussion:**

Our study identifies PLAC1 as a novel potential ligand for NKARs, suggesting it could be a valuable target for pharmacological strategies aimed at enhancing NK cell recognition. This finding holds promise for improving the efficacy of NK cell-based immunotherapies and advancing their clinical application.

## Introduction

Natural killer (NK) are cytolytic and cytokine-producing lymphocytes of the innate immune system, essential for immune regulation and antitumor and antiviral immunity (1). Their activity is tightly controlled by a balance between activating and inhibiting receptors that recognize ligands on the surface of target cells (2).

NK cell activation occurs when inhibitory receptors fail to engage their ligands, while activating receptors bind to their targets, shifting the signaling balance toward activation (3). This interplay determines whether NK cells are triggered to kill target cells (3). NK cell activating receptors (NKARs), such as NK group 2, member D (NKG2D), DNAX accessory molecule 1 (DNAM1) as well as natural cytotoxicity receptors (NCRs) NKp30, NKp44, and NKp46, recognize stress-induced ligands expressed during cellular transformation or viral infection (4). For instance, the homodimer NKG2D detects MICA, MICB, and ULBP family ligands in humans (5), and H60 and Rae1 in mice (6). DNAM-1 recognizes PVR and Nectin-2 (3, 7). Despite initial challenges, several NCR ligands have been identified. NKp30 binds BAT3, HCMV pp65, β-1,3-glucan, and the tumor-associated ligand B7-H6, promoting IFN-γ production and cytotoxicity (8–14). NKp44 and NKp46 bind viral hemagglutinins via sialylated glycans (15–17). NKp44 also recognizes NID1 and tumor-specific MLL5 (18, 19). NKp46 interacts with complement factor P and externalized calreticulin (20, 21).

Although much is known about the expression and function of NKARs, many ligands have not yet been unidentified. To uncover novel ligands for NKARs, we performed a genome-wide loss-of-function screening using stable gene knockdown in the human chronic myeloid leukemia cell line K562 (22, 23). This approach led to the identification of PLAC1 (24) as a potential ligand for NKARs.

Our findings demonstrate that modulating PLAC1 expression, through either inhibition or overexpression, significantly influences NK cell function and their interaction with NKARs such as NKG2D, DNAM-1, NKp44 and NKp30. Notably, PLAC1 is absent in normal tissues under steady-state conditions but is expressed across a wide range of hematopoietic and non-hematopoietic tumors, as well as in transformed cells. In these contexts, PLAC1 can activate allogenic NK cells. Furthermore, its expression has been linked to either favorable or poor prognosis, depending on the tumor type. Altogether, these findings suggest PLAC1 as a promising novel ligand for NKARs.

## Results

### Genome-wide screening identifies PLAC1 as a novel potential ligand for NK cell activating receptors

**Figure 1:**
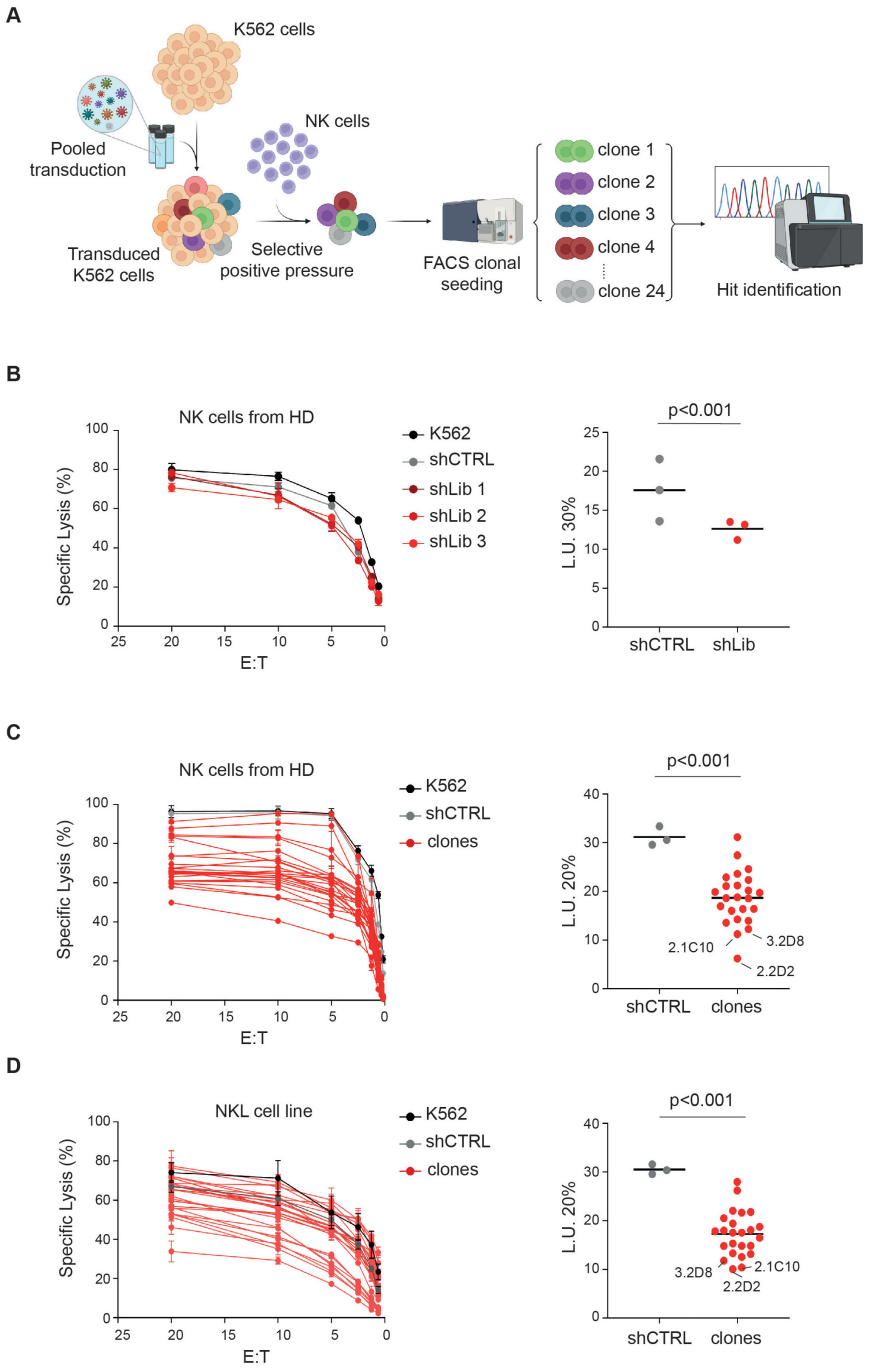
Genome-wide screening identifies new potential ligands for activating NK cell receptors. **(A)** Experimental scheme as detailed in the text. **(B-D)** NK cells derived from HD **(B, C)** and NKL cell line **(D)** were tested as effectors at the indicated E:T ratios in a standard ^51^Cr-release assay using K562 cells infected with the lentiplex shRNA library that survived positive selection with NK cells, either as a polyclonal cell population **(B)** or as single clones **(C, D)**. Data from a representative of three independent experiments are shown. Specific lysis was converted to L.U. 30% **(B)** or 20% **(C, D)**. Dots, represent L.U. 30% or 20% of the effector/target pairs tested; horizontal bars indicate average values. *P* values were calculated by comparing with shCTRL and sh-bulk **(B)** or clones **(C, D)** (two-tailed paired Student *t* test). The scheme in A was created by BioRender.

To uncover novel ligands for NKARs, we performed a genome-wide loss-of-function genetic screening using a pooled shRNA library targeting 15,000 human genes with multiple sequence-verified constructs (**Figure 1A**). On average, five shRNA designs were used per gene. We selected the K562 cell line as a target due to its expression of numerous ligands for NKARs, making it an ideal model for identifying genes involved in NK cell activation. K562 cells were transduced with the human lentiplex shRNA library (TRC 1.5) at a multiplicity of infection (MOI) ≤1 and selected with puromycin for 4 days. As a control, K562 cells were transduced with a non-targeting shRNA (shCTRL). The transduced cells were then cocultured with freshly isolated NK cells from five healthy donors (HD) at an effector-to-target (E:T) ratio of 15:1, with each donor added separately over three consecutive days. Surviving K562 cells, presumed to have silenced genes essential for NK cell-mediated activation, were selected based on viability and morphology via flow cytometry, and maintained as bulk populations or individual clones (**Figure 1A**).

Functional validation using NK cells from additional HDs and a standard ^51^Cr release assay confirmed that K562 cells transduced with the shRNA library were more resistant to NK cell-mediated killing, compared to shCTRL cells (**Figures 1B, C**). The average lytic units (L.U.) at 30% lysis across three replicates (shLib 1-3) were significantly lower for K562-shLib cells (12.6) than for K562-shCTRL cells (17.5) (**Figure 1B**, right panel). Similarly, individual clones showed variable levels of resistance, with library-derived clones displaying an average L.U. at 20% of 18, compared to 31 for shCTRL cells (**Figure 1C**, right panel). Notably, the 10 clones with L.U. values below the average (<18) were also more resistant to killing by the NKL cell line (25) than the other clones (**Figure 1D**).

To evaluate whether silencing a specific ligand could impair NK cell function, we tested NKL cell recognition of K562 cells with MICA knockdown, a known ligand for NKG2D. K562-shMICA cells exhibited protection levels comparable to the top 10 resistant clones, with a L.U. of 40 versus 20 for K562-shCTRL (**Supplementary Figure S1**).

DNA sequencing identified the silenced target genes in 15 out of 24 clones (**Supplementary Table S1**). The remaining 9 clones likely contained multiple shRNA inserts, preventing target identification. Among the identified genes, placenta-specific protein 1 (*PLAC1*, clone 2.2D2) (24), transcription factor 7 (*TCF7*, clone 2.1C10) (26), and Pleckstrin Homology Domain-Containing A5 (*PLEKHA5*, clone 3.2D8) (27) conferred strong resistance to NK cell-mediated lysis by both HD-derived NK cells and the NKL cell line (**Figures 1C, D**). *PLAC1* is associated with placental development (24), *TCF7* is involved in T cell differentiation (28), and *PLEKHA5* has been linked to the suppression of tumor metastasis (29).

**Figure 2:**
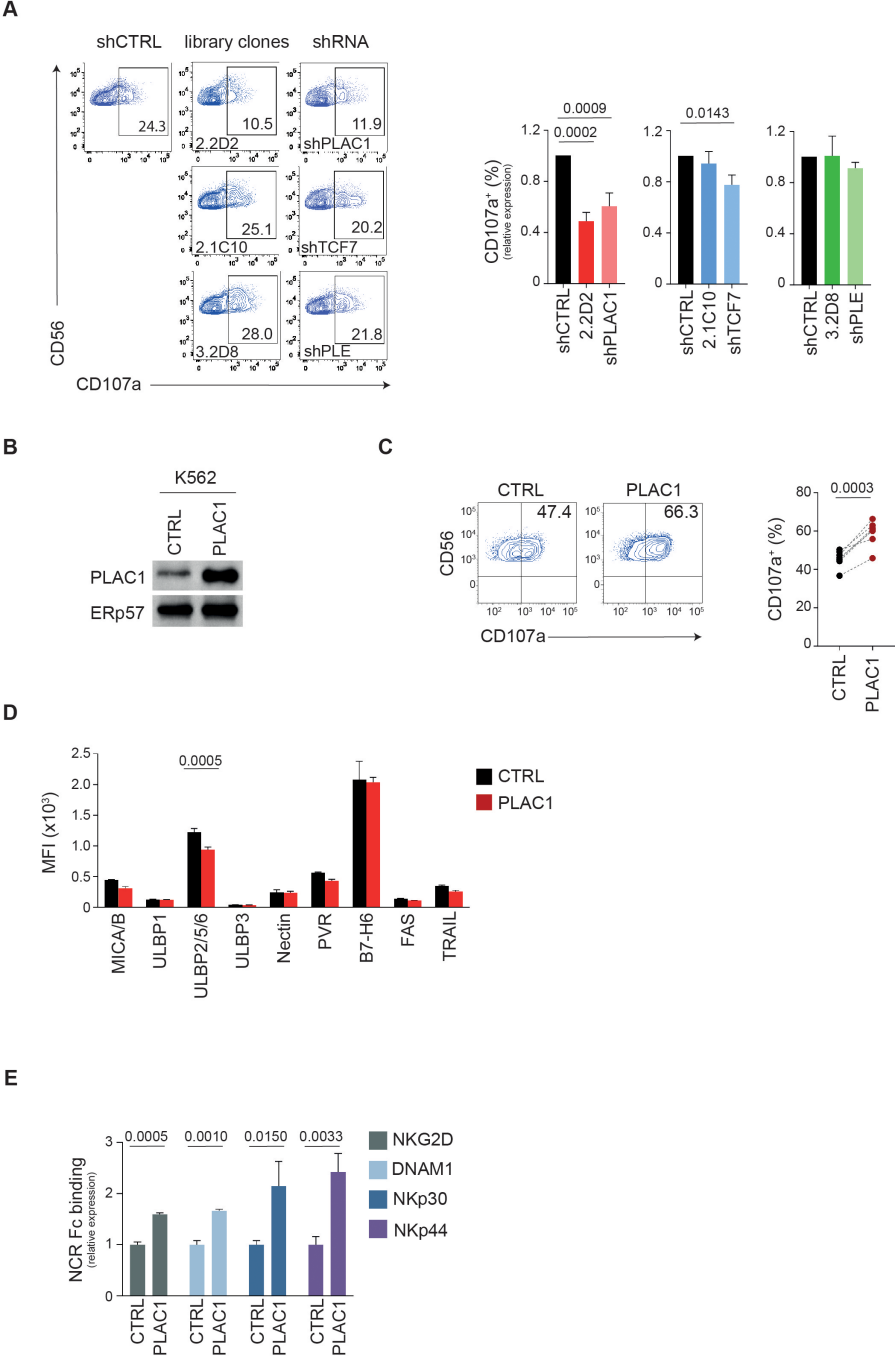
PLAC1 regulates the activity of NK cells. **(A)** Representative example of degranulation by human CD3^−^CD56^+^ NK cells from a HD, measured as CD107a cell-surface expression following stimulation with K562 cells transduced with the indicated library clones or specific shRNAs. The percentage of CD107a^+^ NK cells is indicated. A representative of three independent experiments is reported. Summary of NK cell degranulation after stimulation with the indicated target cells with 5 HDs is reported on the right (Dunett’s multiple comparisons test). **(B)** Immunoblotting of PLAC1 expression in K562 cells overexpressing PLAC1. **(C)** Representative example of degranulation by human CD3^−^CD56^+^ NK cells from a HD, measured as CD107a cell-surface expression following stimulation with K562 cells overexpressing PLAC1. Summary of NK cell degranulation of 7 donors after stimulation with the indicated cells is reported on the right (two-tailed paired Student *t* test). **(D)** Expression of the indicated ligands for NKARs in control and PLAC1-overexpressing K562 cells (Šídák’s multiple comparisons test). **(E)** Relative intensity in NKAR-Fc protein binding on K562 overexpressing PLAC1 compared with K562-CTRL cells measured by flow cytometric analysis. Means ± SD of three independent experiments are shown (two-tailed unpaired Student *t* test).

Functional validation revealed that silencing PLAC1 significantly impaired NK cell degranulation, as indicated by reduced CD107a expression (**Figure 2A**; **Supplementary Figure S2A**). Both K562-shPLAC1 cells and the corresponding library clone (K562-2.2D2) decreased the frequency of CD107a^+^ NK cells by nearly 50% compared to K562-shCTRL cells (0.60 and 0.48, respectively) (**Figure 2A**). In contrast, silencing TCF7 or PLEKHA5 had no significant effect on NK cell degranulation (**Figure 2A**). Based on these results and given its predicted membrane localization (30), we focused on PLAC1 as the most promising target. To further validate its role, PLAC1 was cloned from JEG3 cells and stably expressed in K562 cells (**Figure 2B**; **Supplementary Figures S2B, C**).

Overexpression of PLAC1 in K562 cells increased NK cell degranulation by 23%, with the frequency of CD107a^+^ NK cells rising from 45.5% in K562-CTRL cultures to 59% in K562-PLAC1 cultures (**Figure 2C**). Since the overexpression of PLAC1 did not impact the expression of NKAR ligands MICA/B, ULBPs, Nectin-2, PVR, B7-H6, FAS and TRAIL-R2 (**Figure 2D**), the increased degranulation was attributed to a direct effect mediated by PLAC1.

These results highlight PLAC1’s critical role in modulating NK cell function and suggest its potential as a novel ligand for NKARs.

### PLAC1 modulates the binding of NK cell activating receptors to K562 cells

PLAC1 may influence NK cell function by serving as a ligand for NKARs. To test this hypothesis, we examined whether PLAC1 expression affects the binding of NKARs fusion proteins NKG2D, DNAM1, NKp30 and NKp44 to K562 cells overexpressing PLAC1. In this assay, K562 cells were stained with NKARs-Fc fusion proteins, NKG2D-Fc, DNAM1-Fc, NKp30-Fc and NKp44-Fc. Compared with control cells, overexpression of PLAC1 led to significantly increased binding of all fusion proteins tested in K562 cells (**Figure 2E**).

These results suggest that overexpression of PLAC1 results in increased interaction of these cells with the activating receptors NKG2D, DNAM1, NKp30, and NKp44, potentially amplifying the recognition and lysis of tumor cells by NK cells.

### PLAC1 expression in tumors based on cancer genomic datasets

**Figure 3:**
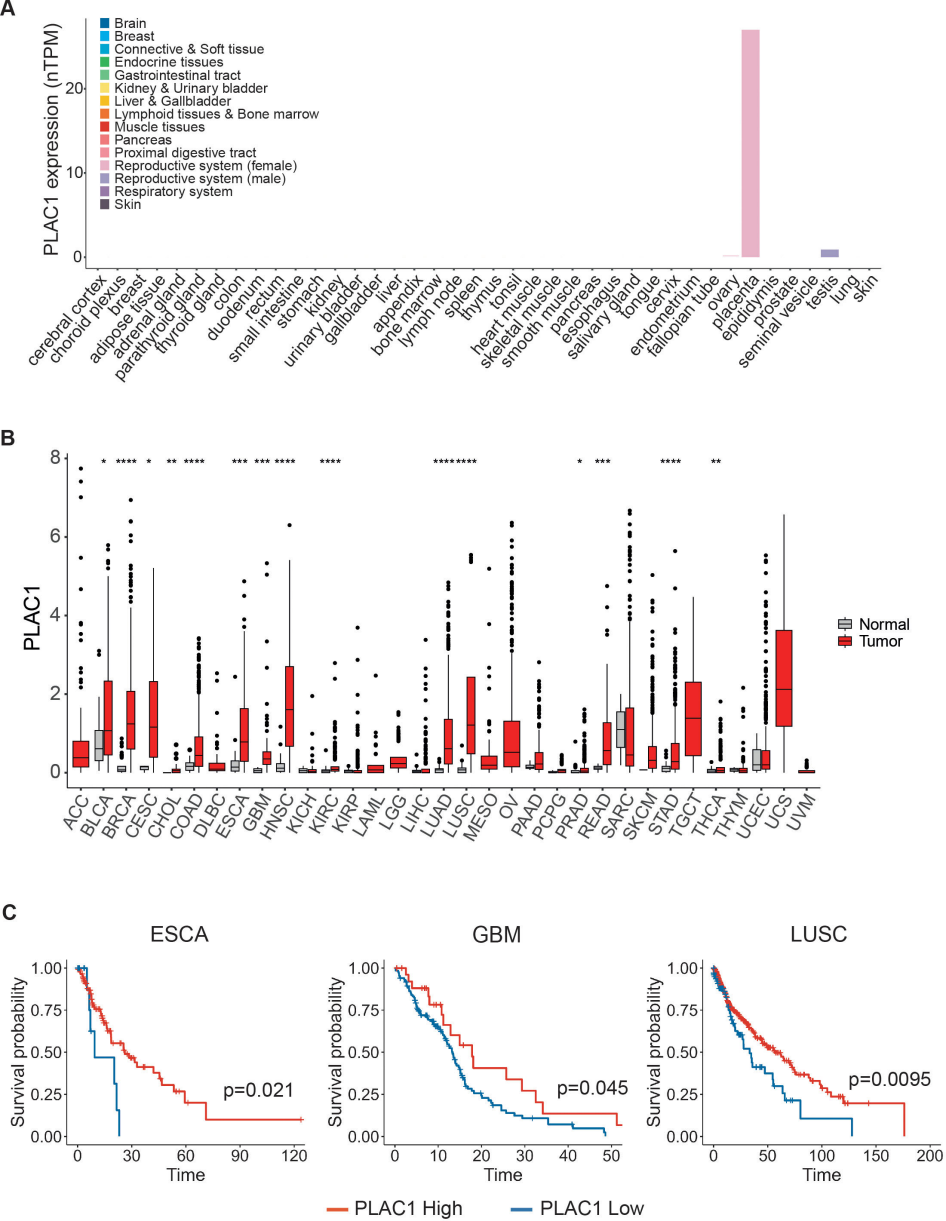
PLAC1 expression has prognostic value in tumors. **(A)** PLAC1 gene expression in the indicated normal human tissues. **(B)** The expression status of PLAC1 in 33 cancer types compared to the normal counterpart. **(C)** Kaplan-Meier curves show the duration of overall survival of the indicated tumor patients according to the PLAC1 gene expression. Statistically significant P values are indicated. * p<0.05, ** p<0.01, *** p<0.001, **** p<0.0001.

PLAC1 is highly expressed during development, particularly in placental tissues, including trophoblast giant cells and the labyrinthine layer derived from the trophoblast lineage (31). According to data from The Cancer Genome Atlas (TCGA), PLAC1 is virtually undetectable in most normal somatic tissues, with the exception of testicular germ cells, skeletal muscle, peritubular cells, and the placenta during gestation (**Figure 3A**; **Supplementary Figure S3A**). In contrast, PLAC1 is significantly upregulated in a wide range of tumor types compared to their normal tissue counterparts (**Figure 3B**). Elevated expression was observed in 15 distinct cancers, including bladder urothelial carcinoma (BLCA), breast invasive carcinoma (BRCA), cervical squamous cell carcinoma and endocervical adenocarcinoma (CESC), cholangiocarcinoma (CHOL), colon adenocarcinoma (COAD), esophageal carcinoma (ESCA), glioblastoma multiforme (GBM), head and neck squamous cell carcinoma (HNSC), kidney renal clear cell carcinoma (KIRC), lung adenocarcinoma (LUAD), lung squamous cell carcinoma (LUSC), prostate adenocarcinoma (PRAD), rectum adenocarcinoma (READ), stomach adenocarcinoma (STAD) and thyroid carcinoma (THCA) (**Figure 3B**; **Supplementary Table S2**). Interestingly, the prognostic relevance of PLAC1 expression appears to be highly tumor-specific. Elevated PLAC1 levels were associated with improved overall survival in ESCA, GBM and LUSC, and poorer prognosis in BLCA, CESC, CHOL, COAD, HNSC, KIRC and PRAD (**Figure 3C**; **Supplementary Figures S3B, C**). To assess the impact of threshold selection on survival outcomes, we conducted sensitivity analyses using multiple clinically and statistically relevant cut-offs—including the mean, median, and quartiles of PLAC1 expression (**Supplementary Figures S3B**). Although the strength and significance of the associations varied depending on the chosen threshold, consistent trends were observed, particularly in ESCA and LUSC, where the mean expression level reliably stratified survival outcomes (**Supplementary Figures S3B, C**).

These findings underscore the importance of careful threshold selection when utilizing PLAC1 as a prognostic biomarker and suggest that PLAC1 may serve as a context-dependent indicator of clinical outcome, with its prognostic value varying substantially across different tumor types.

**Figure 4:**
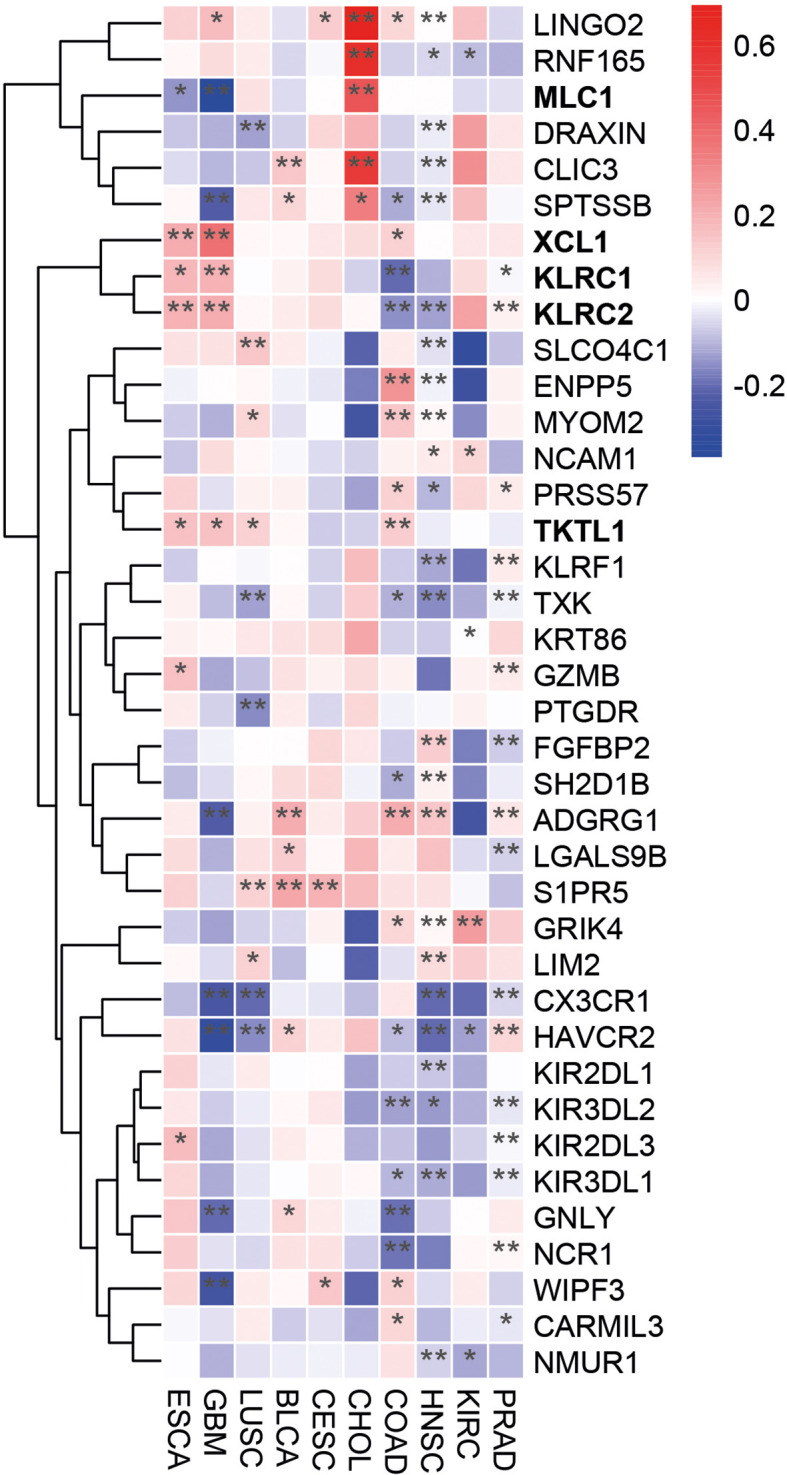
PLAC1 correlates with the expression of NK-cell signature genes in cancer. Heatmap showing the differential expression of NK cell marker genes in the indicated tumor types. The color scale is based on *z*-score-scaled gene expression. The z-score distribution ranges from −2 (blue) to 2 (red). Statistically significant P values are indicated; * p<0.05, ** p<0.01.

To further explore the role of PLAC1 in NK cell activation, we analyzed its correlation with a NK cell gene signature (32) in tumor types where PLAC1 expression affects overall survival of patients. Notably, in tumors where high PLAC1 expression is associated with a favorable prognosis (ESCA, GBM, LUSC), we observed a positive correlation between PLAC1 and the genes *XCL1, KLRC1* (NKG2A*)*, *KLRC2* (NKG2C) and *TKTL1*, and a negative correlation with the *MLC1* gene (**Figure 4**). In contrast, these correlations were absent in tumor types where high PLAC1 expression was associated with poorer survival outcomes (BLCA, CESC, CHOL, COAD, HNSC, KIRC and PRAD). These findings further strengthen the association between PLAC1 and NK cell function in specific cancer contexts.

### Desmoglein 2 is not expressed on NK cells

Recent studies have identified Desmoglein 2 (DSG2), a critical component of desmosomes, as a direct interaction partner of PLAC1 (30). Desmosomes are specialized adhesive protein complexes located at intercellular junctions, essential for maintaining tissue mechanical integrity (33). Given PLAC1’s potential role as a ligand for NKARs, we investigated whether DSG2 is expressed on NK cells. According to data from the Human Protein Atlas (HPA), DSG2 is primarily expressed in epithelial cells and trophoblasts (34). Among immune cells, DSG2 is predominantly found on dendritic cells (DC), with no significant expression detected on NK cells (11) (**Supplementary Figure S4A**). To further explore DSG2 expression in NK cells within an oncological context, we analyzed single-cell RNA sequencing (scRNA-seq) data from NK cells isolated from the bone marrow of HD and patients with acute myeloid leukemia (AML) (35). DSG2 was detected in fewer than 1% of total NK cells (approximately 0.3%), with no significant differences between healthy and AML conditions (**Supplementary Figure S4B**). In contrast, well-characterized NK cell receptors, such as NKp46 (*NCR1*), NKG2D (*KLRK1*), NKG2A (*KLRC1*), and DNAM1 (*CD226*) were expressed in a substantially higher proportion of NK cells, approximately 30%, 10%, 7%, and 1%, respectively (**Supplementary Figure S4B**). Finally, we evaluated the expression of DSG2 on the surface of NK cells purified from HDs. Unlike HeLa cells, which are known to express DSG2, NK cells from all donors analyzed did not express DSG2 (**Supplementary Figure S4C**).

Altogether, these finding indicate that DSG2 is not expressed on the surface of NK cells, neither in healthy conditions nor in cancerous states. Therefore, the interaction between PLAC1 and NK cells likely involves a receptor other than DSG2.

## Discussion

This study identifies a novel role of PLAC1 as a potential ligand for NKARs, contributing to the recognition of tumor cells by NK cells. NKARs, such as NKG2D, DNAM-1 and NCRs NKp30, NKp44 and NKp46, interact with ligands frequently upregulated on tumor cells, thereby promoting NK cell activation, cytokine production and the elimination of tumor cells (1). PLAC1 appears to function similarly, although the precise mechanisms and receptor interactions have yet to be fully elucidated.

The *PLAC1* gene is primarily expressed in the placenta, where it plays a crucial role in fetal development (24). Its protein product is mainly localized on the surface of trophoblastic cells, where it contributes to cell adhesion processes and helps to maintain the maternal-fetal interface (31). Notably, PLAC1 is not expressed in other normal tissues but is detectable in a variety of cancers, making it an attractive target for cancer research due to its restricted expression pattern and immunogenic potential (36–38).

PLAC1 is classified as a cancer/testis antigen (CTA) (39), with elevated expression reported in several tumor types, including stomach (40), colon (41), liver (42), pancreas (43), prostate (44), ovary (45, 46), uterus (47), cervix (48), breast (49), lung (37), HNSC (38, 50) and nasopharynx carcinoma (51). Beyond its expression profile, PLAC1 has been implicated in promoting cancer cell proliferation, invasion, and migration (52). Several factors may explain PLAC1’s role in cancer. For example, it might be ectopically expressed in tumors due to widespread epigenetic deregulation, without directly contributing to oncogenic processes. Its role could be context-dependent, becoming functionally relevant only in certain tumor types, disease stages, or microenvironmental conditions. Alternatively, PLAC1 may participate in broader molecular networks, where its effects are compensated by other mechanisms, making its individual contribution difficult to detect experimentally. Finally, PLAC1 might play a primarily immunological role, contributing to tumor immunogenicity rather than directly driving oncogenic transformation.

In this study, we employed a genome-wide loss-of-function screening approach to demonstrate that PLAC1 can act as a ligand for NKARs. This interaction enhances NK cell-mediated cytotoxicity against PLAC1-expressing tumor cells. Notably, overexpression of PLAC1 in K562 cells increased binding to NKAR-Fc fusion proteins, including NKG2D-Fc, DNAM1-Fc, NKp30-Fc and NKp44-Fc. These findings suggest that PLAC1 may function as a novel ligand for these receptors. Beyond its potential role as a direct ligand for NKARs, PLAC1 could also act as a cofactor for known ligands, modulating their interaction with NKARs through a mechanism distinct from merely inducing ligand expression. While the direct role of PLAC1 as an NKAR ligand remains to be fully validated, our findings underscore its impact on NK cell activation and tumor cell recognition. Further studies are required to determine whether PLAC1 interacts with an as-yet-unidentified NK cell receptor. The potential importance of PLAC1, compared to other tumor-mediated NK cell regulation mechanisms, lies in its role as a direct ligand for NKARs. Unlike stress-induced activation mechanisms such as MICA/B or ULBPs, PLAC1 offers an additional interaction with NK cells, potentially enhancing tumor recognition and killing. This makes PLAC1 a promising target for therapeutic strategies, particularly in tumors that express this antigen.

PLAC1 has been shown to interact with DSG2 in a choriocarcinoma model (30). DSG2 is a key component of desmosomes, structures essential for cell adhesion in epithelial and cardiac tissues (33). As a member of the cadherin superfamily, DSG2 plays diverse roles in cell adhesion and is implicated in both normal development and cancer progression (34, 53). Interestingly, DSG2 is highly expressed in epithelial and cardiac tissues but less so in immune cells, including NK cells, where its expression decreases during lymphocyte differentiation (11). While the role of DSG2 in NK cell function and its potential affinity for PLAC1 warrant further investigation, our data suggests that the interaction between PLAC1 and NK cells likely involves a receptor other than DSG2. Exploring the targeting of PLAC1 to enhance NK cell-mediated anti-tumor responses could lead to the development of new immunotherapies that increase NK cell cytotoxicity or harness PLAC1’s immunogenic potential to improve cancer treatment outcomes. Further studies are needed to assess how PLAC1 influences tumor progression and whether targeting it can effectively modulate the tumor microenvironment.

In summary, the interaction between PLAC1 and NK cells represents a promising mechanism by which the immune system can recognize and eliminate PLAC1-expressing tumors. These findings provide new insights into tumor immunity and suggest potential strategies for cancer immunotherapy involving the modulation of PLAC1. Further studies are needed to unravel the precise molecular pathways and to assess the therapeutic potential of PLAC1 in clinical settings.

## Methods

### Cell lines, patients, NK cells and reagents

All cell lines were obtained by ATCC and characterized every 6 months by HLA class I typing. Mycoplasma contamination was routinely detected by Mycoplasma Detection kit (Venor-GeM Advance). Cells were maintained in RPMI 1640 medium supplemented with 10% FBS (Gibco), 2 mM glutamine, 100 mg/ml penicillin and 50 mg/ml streptomycin (Euroclone).

Human NK cells were isolated from peripheral blood mononuclear cells (PBMC) of HDs by the RosetteSep NK-cell enrichment mixture method (StemCell Technologies) and Ficoll-Paque Plus (Lympholyte Cedarlane) centrifugation. NK cells with purity greater than 90% were suspended in NK MACS medium (Miltenyi Biotec) supplemented with NK MACS Supplement, AB serum and 500 IU/mL of recombinant human IL-2 (PeproTech) for 18 hours at 37°C.

### Lentiviral infections and transfections

K562 cells were stable infected with a pooled shRNA human library from the RNAi consortium (TRC 1.5) consisting of over 150000 plasmid-based shRNA constructs targeting 15000 human genes (Sigma). On average, there were five shRNA designs for each gene target. K562 were stably transduced with a multiplicity of infection (MOI) ≤ 1 such that the majority of cells will be transduced with a single shRNA. Transduced cells were selected using 3 µg/ml puromycin for 4 days. As control K562 cell were infected with an shRNA (sgCTR). Transduced cells were cocultured with NK cells isolated from HD at an E:T ratio of 15:1 for 3 days. The surviving cells were FACS sorted for cell viability and morphology and seeded clonally in 96 well plates. The clones obtained were expanded and genomic DNA extracted by standard procedures. Lentiviral particles were generated in HEK293T cells by combining a pLKO.1 plasmid containing shRNA sequences, packaging plasmid pCMV-dR8.74, and envelope plasmid VSV-G/pMD2.G using TransIT-293 transfection reagent (MIRUS Bio LLC, Madison, WI, USA). K562 cells were infected by the spin inoculation method with lentivirus containing a nontarget shRNA control vector (SHC002) or either of MICA, PLAC1, TCF7 and PLEK shRNAs (clone ID: TRCN0000061288, TRCN0000061031, TRCN0000061677, and TRCN0000060846) targeting the indicated genes (Sigma-Aldrich).

### PLAC1 cloning

Full-length cDNAs encoding human PLAC1 (accession number AF234654.1) was cloned from JEG3 cells in the lentiviral vector pRRL-CMV-PGK-GFP-WPRE (TWEEN) under the control of the CMV promoter. K562 cells were transfected with PLAC1 vector or empty vector using LipofectAMINE 2000 according to the manufacturer’s instructions (Invitrogen Life Technologies). Stably transfected cells were sorted by FACS as GFP expressing cells.

### DNA sequencing

Genomic DNA was isolated with standard procedures and primers designed to flank the shRNA hairpins were used to amplify the shRNA sequences. Hits identified by sequencing the PCR amplicon were matched to database.

### Quantitative mRNA expression

Total RNA was extracted using TRIzol Reagent (Invitrogen). First-strand cDNA was synthesized using the SuperScript II First Strand cDNA synthesis kit (Invitrogen). Quantitative real-time PCR (qPCR) reactions were performed using pre-validated TaqMan gene expression assays from Applied Biosystems (Hs06598311_m1 for PLAC1, Hs01556515_m1 for TCF7, Hs01043767_m1 for PLEKHA5, Hs02786624_g1 for GAPDH). Relative gene expression was determined using the 2-ΔΔCt method with ß-actin as endogenous control.

### Cytotoxicity and degranulation assay

NK cell cytotoxic activity and degranulation assay were performed by a standard 4-hour ^51^Cr-release assay and flow cytometric analysis of cell-surface CD107a expression, respectively. In cytotoxicity assay K562 cells were labelled with ^51^Cr [Amersham International; 100μCi (3.7 MBq)/1 x 10^6^ cells] and co-cultured (5 x 10^3^) with NK cells or NKL cell line at different E:T cell ratios, in 96-well plates round bottom in triplicates, and incubated at 37°C. At 4 hours of incubation, 25μL supernatant were removed, and the ^51^Cr release was measured with TopCount NXT beta detector (PerkinElmer Life Sciences). The percentage of specific lysis by counts per minute (cpm) was determined as follows: 100 x (mean cpm experimental release – mean cpm spontaneous release)/(mean cpm total release - mean cpm spontaneous release). Specific lysis was converted to lytic units (L.U.) calculated from the curve of the percentage lysis (54) and defined as the number of NK cells required to produce 20% lysis of 10^6^ target cells during the 4 hours of incubation. In degranulation assays, NK cells were co-cultured with K562 target cells at 1:1 ratio for 3 hours, in complete medium in the presence of anti-CD107a (diluted 1:100). During the last 2 hours, GolgiStop (BD Bioscences) was added at 1:500 dilution. Cells were firstly pre-stained with Live/Dead Kit (L/D), stained with anti-CD56, anti-CD3 and then, the expression of CD107a was evaluated in the CD3^-^CD56^+^ subset by flow cytometry.

### Antibodies for flow cytometry

The following antibodies were used: anti-CD3-Alexa-700 (UCHT1), anti-CD56-PE-Cy7 (B159), anti-CD107a-FITC (H4A3), anti-IgG1-FITC (A85-1), anti-IgM-PE (R6-60.2), anti-MICA/B-BV650 (6D4), anti-ULBP2/5/6-BV510 (165903), anti-CD155/PVR-BV605 (SKII.4), anti-CD112/Nectin-2-PE (R2.525), anti-CD95/FAS-BV421 (DX2), anti-CD262/TRAIL/R2-PE (YM366), anti-ULBP1-PE (170818), anti-ULBP3-PE (166510), anti-FAS-BV421 (DX2), anti-B7-H6-BV421 (1A5), anti-DSG2-PE (6D8), purchased from BD Biosciences. For indirect staining, goat F(ab’)2 Fragment anti-mouse IgG FITC (IM1619) was used. All these antibodies were used according to the manufacturers’ protocol. Prior to surface staining, NK cells were pre-stained with Live/Dead™ Fixable Near-IR Dead Cell Stain Kit (Invitrogen). Flow cytometry was performed by using FACSCalibur, FACSCantoII or FACSFortessa X-20 (BD Biosciences) and analyzed by FlowJo Software. Recombinant human NKG2D-Fc, DNAM1-Fc, NKp30-Fc and NKp44-Fc chimera proteins were purchased from R&D. Cells were incubated with fusion proteins for 30 min in ice, washed twice and then incubated with PE-conjugated goat anti-human Fc antibody (Jackson ImmunoResearch Laboratories) for 30 min on ice, and propidium iodide was added to cells before flow analysis.

### Western blotting

Equal amounts of protein extracts were resolved on 15% or 10% polyacrylamide gel for the detection of PLAC1 and MICA, respectively, and transferred on nitrocellulose membranes (Amersham Systems, Ge Healthcare Sciences). Filters were blocked with 5% (v/v) non-fat dry milk for 1 hour at room temperature, and then blotted with rabbit anti-human PLAC1 polyclonal antibody (kindly provided by Prof. Zarnani, Sina Biotech Co) or mouse anti-human PLAC1 (G1, Santa Cruz Biotechnology, Cat No 365919), and mouse anti-human MICA monoclonal antibody (Proteintech, Cat No. 66384-1-Ig) to recognize PLAC1 and MICA, respectively. Anti-ERp57, anti-β−actin or anti-GAPDH antibodies were used as loading control. After extensive washing with TBST, filters were incubated with peroxidase-coupled secondary antibody for 1 hour at room temperature. Reactivity was detected with the ECL Western Blotting Detection Kit (Amersham Systems, Ge Healthcare Life Sciences) and the protein bands were quantified using Image J.

### Databases and data analysis

Pan-cancer gene expression data and clinical data from 33 tumor types were downloaded from The Cancer Genome Atlas (TCGA, https://portal.gdc.cancer.gov/, accessed April 5^th^, 2024) database. The survival curves using log-rank test of Kaplan-Meier overall survival (OS) and the forest-plots indicating hazard-ratio and 95% confidence intervals for different cut-off values for PLAC1 expression were conducted using the *survival* R package (version 3.4.0) under R version 4.4.1 (https://www.R-project.org/). P values<0.05 were considered significant. The expression of PLAC1 and DSG2 in healthy tissues and cell types was obtained from the Human Protein Atlas (HPA, http://www.proteinatlas.org/, accessed July 2^nd^, 2024) database. The Gene Expression Omnibus (GEO, https://www.ncbi.nlm.nih.gov/geo/, accessed September 6^th^, 2024) database was used to obtain normalized scRNA-seq data from NK cells (GSE159624).

### Statistical analysis

For all data, statistical significance was evaluated using GraphPad software. Statistical tests performed are indicated in the figure legends. P values not exceeding 0.05 were considered to be statistically significant.

## Data Availability

The original contributions presented in the study are included in the article/**Supplementary Material**. Further inquiries can be directed to the corresponding author.

## Supplementary material

Supplementary Figure 1 Related to **Figure 1**. MICA inhibition confers protection of K562 cells from NK cell-mediated lysis. **(A)** Representative immunoblotting analysis of MICA expression on K562 cells infected with lentiviruses carrying control shRNA (shMICA-) or shRNA targeting the *MICA* gene (shMICA+). Densitometric analysis of GAPDH-normalized MICA expression from three independent experiments is shown below. **(B)** Flow-cytometry analysis of MICA expression in the indicated cell lines. The percentage of MICA-positive K562 cells is shown. **(C)** K562-shMICA and K562-shCTRL cells were assayed as targets of NK cells at the indicated E:T ratios in a standard ^51^Cr-release assay. A representative of five independent experiments is reported. **(D)** Specific lysis of C was converted to L.U. 20%. Dots, L.U. 20% of the effector/target pairs tested; horizontal bars, average values. *P* values, compared with K562-shMICA and K562-shCTRL cells (two-tailed paired Student *t* test).

Supplementary Figure 2 Related to **Figure 2**. PLAC1 regulates the activity of NK cells. **(A)** qPCR of PLAC1, TCF7 and PLEKHA5 expression in K562 cells transduced with lentiviral vectors encoding either control shRNA (shCTRL) or shRNA targeting PLAC1, TCF7 or PLEKHA5 genes (two-tailed unpaired Student *t* test). **(B)** qPCR of PLAC1 expression in K562 cells overexpressing PLAC1 (two-tailed unpaired Student *t* test).

Supplementary Figure 3 Related to **Figure 3** PLAC1 expression has prognostic value in tumors. **(A)** PLAC1 gene expression in the indicated human cell types**. (B)** Kaplan-Meier curves show the duration of overall survival of the indicated tumor patients according to the PLAC1 gene expression. **(C)** Forest plots showing hazard ratio and 95% confident intervals of the full range of cutoff values of the indicated tumor patients according to the PLAC1 gene expression. Log-rank test with Miller and Siegmund P-value correction was used. Statistically significant P values are indicated.

Supplementary Figure 4 Desmoglein 2 expression in tumor cells and NK cells. **(A)** DSG2 expression in the indicated human cells. **(B)** Density plots of DSG2 expression of NK cells from scRNA-seq of HD and AML patients (GSE159624). **(C)** Representative flow-cytometry analyses of DSG2 expression in HeLa cells and NK cells from a HD. Summary of DSG2 expression in NK cells from 3 HDs is reported on the right.

## References

[1] Quatrini L Della Chiesa M Sivori S Mingari MC Pende D Moretta L. Human NK cells, their receptors and function. Eur J Immunol. (2021) 51:1566–79. doi: 10.1002/eji.202049028 PMC9292411 33899224

[2] Lanier LL. Up on the tightrope: natural killer cell activation and inhibition. Nat Immunol. (2008) 9:495–502. doi: 10.1038/ni1581 18425106 PMC2669298

[3] Biassoni R Cantoni C Pende D Sivori S Parolini S Vitale M. Human natural killer cell receptors and co-receptors. Immunol Rev. (2001) 181:203–14. doi: 10.1034/j.1600-065X.2001.1810117.x 11513142

[4] Lanier LL. NK cell receptors. Annu Rev Immunol. (1998) 16:359–93. doi: 10.1146/annurev.immunol.16.1.359 9597134

[5] Zingoni A Molfetta R Fionda C Soriani A Paolini R Cippitelli M. NKG2D and its ligands: “One for all, all for one. Front Immunol. (2018) 9:476. doi: 10.3389/fimmu.2018.00476 29662484 PMC5890157

[6] Gonzalez S Lopez-Soto A Suarez-Alvarez B Lopez-Vazquez A Lopez-Larrea C. NKG2D ligands: key targets of the immune response. Trends Immunol. (2008) 29:397–403. doi: 10.1016/j.it.2008.04.007 18602338

[7] Cifaldi L Doria M Cotugno N Zicari S Cancrini C Palma P. DNAM-1 activating receptor and its ligands: how do viruses affect the NK cell-mediated immune surveillance during the various phases of infection? Int J Mol Sci. (2019) 20. doi: 10.3390/ijms20153715 PMC6695959 31366013

[8] Pogge von Strandmann E Simhadri VR von Tresckow B Sasse S Reiners KS Hansen HP. Human leukocyte antigen-B-associated transcript 3 is released from tumor cells and engages the NKp30 receptor on natural killer cells. Immunity. (2007) 27:965–74. doi: 10.1016/j.immuni.2007.10.010 18055229

[9] Arnon TI Achdout H Levi O Markel G Saleh N Katz G. Inhibition of the NKp30 activating receptor by pp65 of human cytomegalovirus. Nat Immunol. (2005) 6:515–23. doi: 10.1038/ni1190 15821739

[10] Li SS Ogbomo H Mansour MK Xiang RF Szabo L Munro F. Identification of the fungal ligand triggering cytotoxic PRR-mediated NK cell killing of Cryptococcus and Candida. Nat Commun. (2018) 9:751. doi: 10.1038/s41467-018-03014-4 29467448 PMC5821813

[11] Bagger FO Kinalis S Rapin N. BloodSpot: a database of healthy and Malignant haematopoiesis updated with purified and single cell mRNA sequencing profiles. Nucleic Acids Res. (2019) 47:D881–5. doi: 10.1093/nar/gky1076 PMC6323996 30395307

[12] Brandt CS Baratin M Yi EC Kennedy J Gao Z Fox B. The B7 family member B7-H6 is a tumor cell ligand for the activating natural killer cell receptor NKp30 in humans. J Exp Med. (2009) 206:1495–503. doi: 10.1084/jem.20090681 PMC2715080 19528259

[13] Cao G Wang J Zheng X Wei H Tian Z Sun R. Tumor therapeutics work as stress inducers to enhance tumor sensitivity to natural killer (NK) cell cytolysis by up-regulating NKp30 ligand B7-H6. J Biol Chem. (2015) 290:29964–73. doi: 10.1074/jbc.M115.674010 PMC4705966 26472927

[14] Semeraro M Rusakiewicz S Minard-Colin V Delahaye NF Enot D Vely F. Clinical impact of the NKp30/B7-H6 axis in high-risk neuroblastoma patients. Sci Transl Med. (2015) 7:283ra255. doi: 10.1126/scitranslmed.aaa2327 25877893

[15] Mandelboim O Lieberman N Lev M Paul L Arnon TI Bushkin Y. Recognition of haemagglutinins on virus-infected cells by NKp46 activates lysis by human NK cells. Nature. (2001) 409:1055–60. doi: 10.1038/35059110 11234016

[16] Ho JW Hershkovitz O Peiris M Zilka A Bar-Ilan A Nal B. H5-type influenza virus hemagglutinin is functionally recognized by the natural killer-activating receptor NKp44. J Virol. (2008) 82:2028–32. doi: 10.1128/JVI.02065-07 PMC2258730 18077718

[17] Dong XY Peng JR Ye YJ Chen HS Zhang LJ Pang XW. Plac1 is a tumor-specific antigen capable of eliciting spontaneous antibody responses in human cancer patients. Int J Cancer. (2008) 122:2038–43. doi: 10.1002/ijc.v122:9 18183594

[18] Gaggero S Bruschi M Petretto A Parodi M Del Zotto G Lavarello C. Nidogen-1 is a novel extracellular ligand for the NKp44 activating receptor. Oncoimmunology. (2018) 7:e1470730. doi: 10.1080/2162402X.2018.1470730 30228939 PMC6140582

[19] Baychelier F Sennepin A Ermonval M Dorgham K Debre P Vieillard V. Identification of a cellular ligand for the natural cytotoxicity receptor NKp44. Blood. (2013) 122:2935–42. doi: 10.1182/blood-2013-03-489054 23958951

[20] Narni-Mancinelli E Gauthier L Baratin M Guia S Fenis A Deghmane AE. Complement factor P is a ligand for the natural killer cell-activating receptor NKp46. Sci Immunol. (2017) 2. doi: 10.1126/sciimmunol.aam9628 PMC5419422 28480349

[21] Sen Santara S Lee DJ Crespo A Hu JJ Walker C Ma X. The NK cell receptor NKp46 recognizes ecto-calreticulin on ER-stressed cells. Nature. (2023) 616:348–56. doi: 10.1038/s41586-023-05912-0 PMC10165876 37020026

[22] Zamai L Mariani AR Zauli G Rodella L Rezzani R Manzoli FA. Kinetics of *in vitro* natural killer activity against K562 cells as detected by flow cytometry. Cytometry. (1998) 32:280–5. doi: 10.1002/(SICI)1097-0320(19980801)32:4<280::AID-CYTO4>3.0.CO;2-M 9701396

[23] Diehl P Tedesco D Chenchik A. Use of RNAi screens to uncover resistance mechanisms in cancer cells and identify synthetic lethal interactions. Drug Discov Today Technol. (2014) 11:11–8. doi: 10.1016/j.ddtec.2013.12.002 PMC4031443 24847648

[24] Cocchia M Huber R Pantano S Chen EY Ma P Forabosco A. PLAC1, an Xq26 gene with placenta-specific expression. Genomics. (2000) 68:305–12. doi: 10.1006/geno.2000.6302 10995572

[25] Robertson MJ Cochran KJ Cameron C Le JM Tantravahi R Ritz J. Characterization of a cell line, NKL, derived from an aggressive human natural killer cell leukemia. Exp Hematol. (1996) 24:406–15. 8599969

[26] Zhu Y Wang W Wang X. Roles of transcriptional factor 7 in production of inflammatory factors for lung diseases. J Transl Med. (2015) 13:273. doi: 10.1186/s12967-015-0617-7 26289446 PMC4543455

[27] Tran TT Rane CK Zito CR Weiss SA Jessel S Lucca L. Clinical significance of PDCD4 in melanoma by subcellular expression and in tumor-associated immune cells. Cancers (Basel). (2021) 13. doi: 10.3390/cancers13051049 PMC7958624 33801444

[28] Zhang J Lyu T Cao Y Feng H. Role of TCF-1 in differentiation, exhaustion, and memory of CD8(+) T cells: A review. FASEB J. (2021) 35:e21549. doi: 10.1096/fj.202002566R 33913198

[29] Liu J Adhav R Miao K Su SM Mo L Chan UI. Characterization of BRCA1-deficient premalignant tissues and cancers identifies Plekha5 as a tumor metastasis suppressor. Nat Commun. (2020) 11:4875. doi: 10.1038/s41467-020-18637-9 32978388 PMC7519681

[30] Chen Y Stagg C Schlessinger D Nagaraja R. PLAC1 affects cell to cell communication by interacting with the desmosome complex. Placenta. (2021) 110:39–45. doi: 10.1016/j.placenta.2021.06.001 34118612 PMC8237484

[31] Jackman SM Kong X Fant ME. Plac1 (placenta-specific 1) is essential for normal placental and embryonic development. Mol Reprod Dev. (2012) 79:564–72. doi: 10.1002/mrd.22062 PMC4594876 22729990

[32] Shembrey C Foroutan M Hollande F. A new natural killer cell-specific gene signature predicting recurrence in colorectal cancer patients. Front Immunol. (2022) 13:1011247. doi: 10.3389/fimmu.2022.1011247 36685584 PMC9853446

[33] Brooke MA Nitoiu D Kelsell DP. Cell-cell connectivity: desmosomes and disease. J Pathol. (2012) 226:158–71. doi: 10.1002/path.v226.2 21989576

[34] Awad MM Dalal D Cho E Amat-Alarcon N James C Tichnell C. DSG2 mutations contribute to arrhythmogenic right ventricular dysplasia/cardiomyopathy. Am J Hum Genet. (2006) 79:136–42. doi: 10.1086/504393 PMC1474134 16773573

[35] Crinier A Dumas PY Escaliere B Piperoglou C Gil L Villacreces A. Single-cell profiling reveals the trajectories of natural killer cell differentiation in bone marrow and a stress signature induced by acute myeloid leukemia. Cell Mol Immunol. (2021) 18:1290–304. doi: 10.1038/s41423-020-00574-8 PMC8093261 33239726

[36] Koslowski M Sahin U Mitnacht-Kraus R Seitz G Huber C Tureci O. A placenta-specific gene ectopically activated in many human cancers is essentially involved in Malignant cell processes. Cancer Res. (2007) 67:9528–34. doi: 10.1158/0008-5472.CAN-07-1350 17909063

[37] Yang L Zha TQ He X Chen L Zhu Q Wu WB. Placenta-specific protein 1 promotes cell proliferation and invasion in non-small cell lung cancer. Oncol Rep. (2018) 39:53–60. 29138842 10.3892/or.2017.6086 PMC5783604

[38] Meng X Liu Z Zhang L He Y. Plac1 remodels the tumor immune evasion microenvironment and predicts therapeutic response in head and neck squamous cell carcinoma. Front Oncol. (2022) 12:919436. doi: 10.3389/fonc.2022.919436 35814442 PMC9263085

[39] Li Y Chu J Li J Feng W Yang F Wang Y. Cancer/testis antigen-Plac1 promotes invasion and metastasis of breast cancer through Furin/NICD/PTEN signaling pathway. Mol Oncol. (2018) 12:1233–48. doi: 10.1002/mol2.2018.12.issue-8 PMC6068355 29704427

[40] Liu F Shen D Kang X Zhang C Song Q. New tumour antigen PLAC1/CP1, a potentially useful prognostic marker and immunotherapy target for gastric adenocarcinoma. J Clin Pathol. (2015) 68:913–6. doi: 10.1136/jclinpath-2015-202978 26157147

[41] Ren Y Lv Y Li T Jiang Q. High expression of PLAC1 in colon cancer as a predictor of poor prognosis: A study based on TCGA data. Gene. (2020) 763:145072. doi: 10.1016/j.gene.2020.145072 32827679

[42] Wu Y Lin X Di X Chen Y Zhao H Wang X. Oncogenic function of Plac1 on the proliferation and metastasis in hepatocellular carcinoma cells. Oncol Rep. (2017) 37:465–73. doi: 10.3892/or.2016.5272 27878289

[43] Yin Y Zhu X Huang S Zheng J Zhang M Kong W. Expression and clinical significance of placenta-specific 1 in pancreatic ductal adenocarcinoma. Tumour Biol. (2017) 39:1010428317699131. doi: 10.1177/1010428317699131 28618924

[44] Ghods R Ghahremani MH Madjd Z Asgari M Abolhasani M Tavasoli S. High placenta-specific 1/low prostate-specific antigen expression pattern in high-grade prostate adenocarcinoma. Cancer Immunol Immunother. (2014) 63:1319–27. doi: 10.1007/s00262-014-1594-z PMC11029513 25186610

[45] Tchabo NE Mhawech-Fauceglia P Caballero OL Villella J Beck AF Miliotto AJ. Expression and serum immunoreactivity of developmentally restricted differentiation antigens in epithelial ovarian cancer. Cancer Immun. (2009) 9:6. 19705800 PMC2935768

[46] Devor EJ Gonzalez-Bosquet J Warrier A Reyes HD Ibik NV Schickling BM. p53 mutation status is a primary determinant of placenta-specific protein 1 expression in serous ovarian cancers. Int J Oncol. (2017) 50:1721–8. doi: 10.3892/ijo.2017.3931 PMC5403493 28339050

[47] Devor EJ Leslie KK. The oncoplacental gene placenta-specific protein 1 is highly expressed in endometrial tumors and cell lines. Obstet Gynecol Int. (2013) 2013:807849. doi: 10.1155/2013/807849 23935632 PMC3723095

[48] Devor EJ Reyes HD Gonzalez-Bosquet J Warrier A Kenzie SA Ibik NV. Placenta-specific protein 1 expression in human papillomavirus 16/18-positive cervical cancers is associated with tumor histology. Int J Gynecol Cancer. (2017) 27:784–90. doi: 10.1097/IGC.0000000000000957 PMC5405019 28375929

[49] Yuan H Wang X Shi C Jin L Hu J Zhang A. Plac1 is a key regulator of the inflammatory response and immune tolerance in mammary tumorigenesis. Sci Rep. (2018) 8:5717. doi: 10.1038/s41598-018-24022-w 29632317 PMC5890253

[50] Hayashi R Nagato T Kumai T Ohara K Ohara M Ohkuri T. Expression of placenta-specific 1 and its potential for eliciting anti-tumor helper T-cell responses in head and neck squamous cell carcinoma. Oncoimmunology. (2020) 10:1856545. doi: 10.1080/2162402X.2020.1856545 33457076 PMC7781841

[51] Lin C Qian P Zhang Y Liu Z Dai K Sun D. Plac1 promotes nasopharyngeal carcinoma cells proliferation, migration and invasion via Furin/NICD/PTEN pathway. Tissue Cell. (2021) 69:101480. doi: 10.1016/j.tice.2020.101480 33418237

[52] Mahmoudian J Ghods R Nazari M Jeddi-Tehrani M Ghahremani MH Ghaffari-Tabrizi-Wizsy N. PLAC1: biology and potential application in cancer immunotherapy. Cancer Immunol Immunother. (2019) 68:1039–58. doi: 10.1007/s00262-019-02350-8 PMC11028298 31165204

[53] Myo Min KK Ffrench CB McClure BJ Ortiz M Dorward EL Samuel MS. Desmoglein-2 as a cancer modulator: friend or foe? Front Oncol. (2023) 13:1327478. 38188287 10.3389/fonc.2023.1327478 PMC10766750

[54] Villanueva J Lee S Giannini EH Graham TB Passo MH Filipovich A. Natural killer cell dysfunction is a distinguishing feature of systemic onset juvenile rheumatoid arthritis and macrophage activation syndrome. Arthritis Res Ther. (2005) 7:R30–37. 10.1186/ar1453 PMC1064882 15642140

