# Supplementary figures and images for: A genome-wide shRNA screen uncovers a novel potential ligand for NK cell activating receptors
Source: Front Immunol. 2025 Jun 18;16:1537876. doi: 10.3389/fimmu.2025.1537876 (PMC12213676; doi:10.3389/fimmu.2025.1537876)

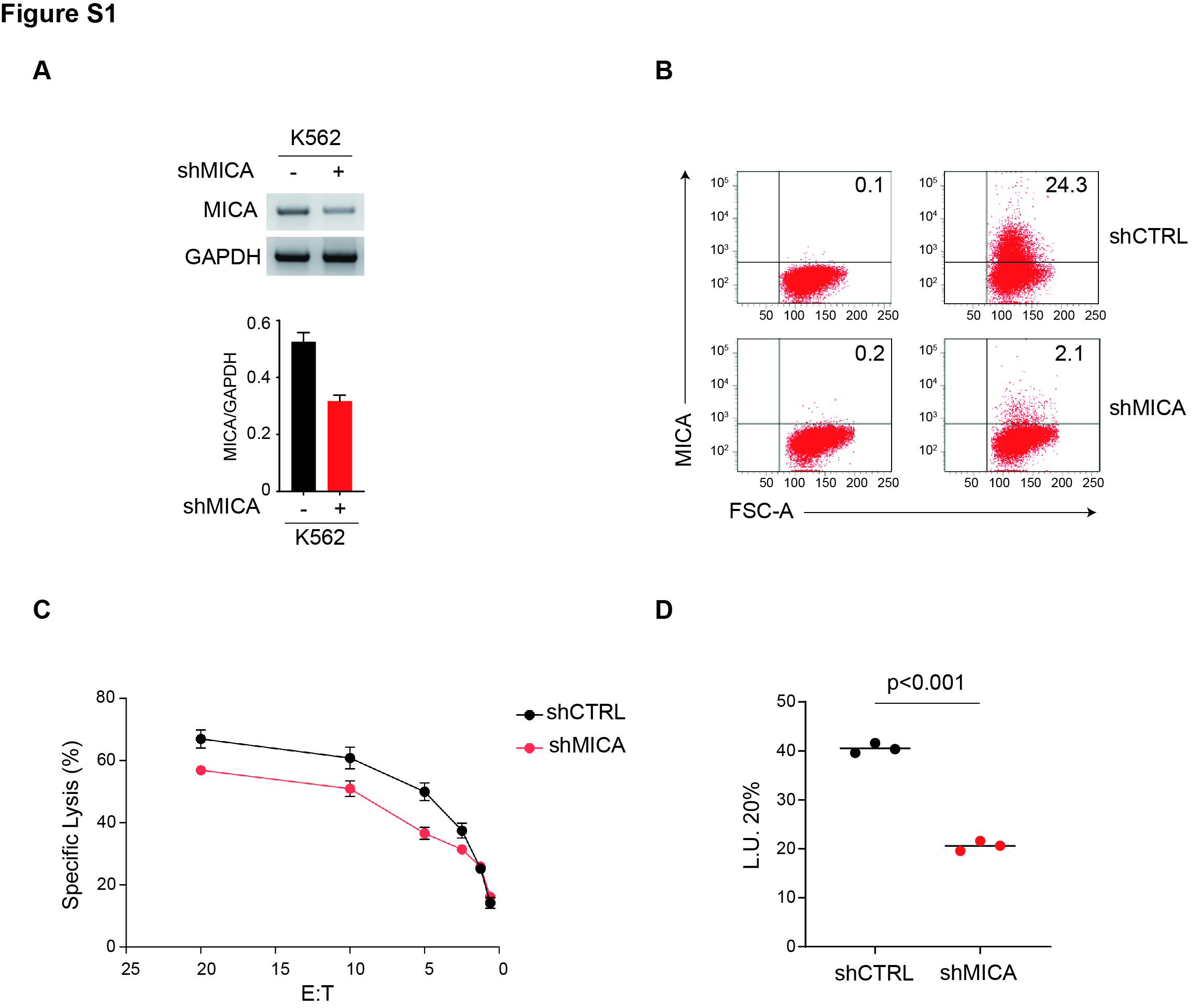

Supplement: Supplementary Figure 1 — Related to Figure 1 . MICA inhibition confers protection of K562 cells from NK cell-mediated lysis. (A) Representative immunoblotting analysis of MICA expression on K562 cells infected with lentiviruses carrying control shRNA (shMICA-) or shRNA targeting the MICA gene (shMICA+). Densitometric analysis of GAPDH-normalized MICA expression from three independent experiments is shown below. (B) Flow-cytometry analysis of MICA expression in the indicated cell lines. The percentage of MICA-positive K562 cells is shown. (C) K562-shMICA and K562-shCTRL cells were assayed as targets of NK cells at the indicated E:T ratios in a standard 51Cr-release assay. A representative of five independent experiments is reported. (D) Specific lysis of C was converted to L.U. 20%. Dots, L.U. 20% of the effector/target pairs tested; horizontal bars, average values. P values, compared with K562-shMICA and K562-shCTRL cells (two-tailed paired Student t test). [file Image1.jpg]

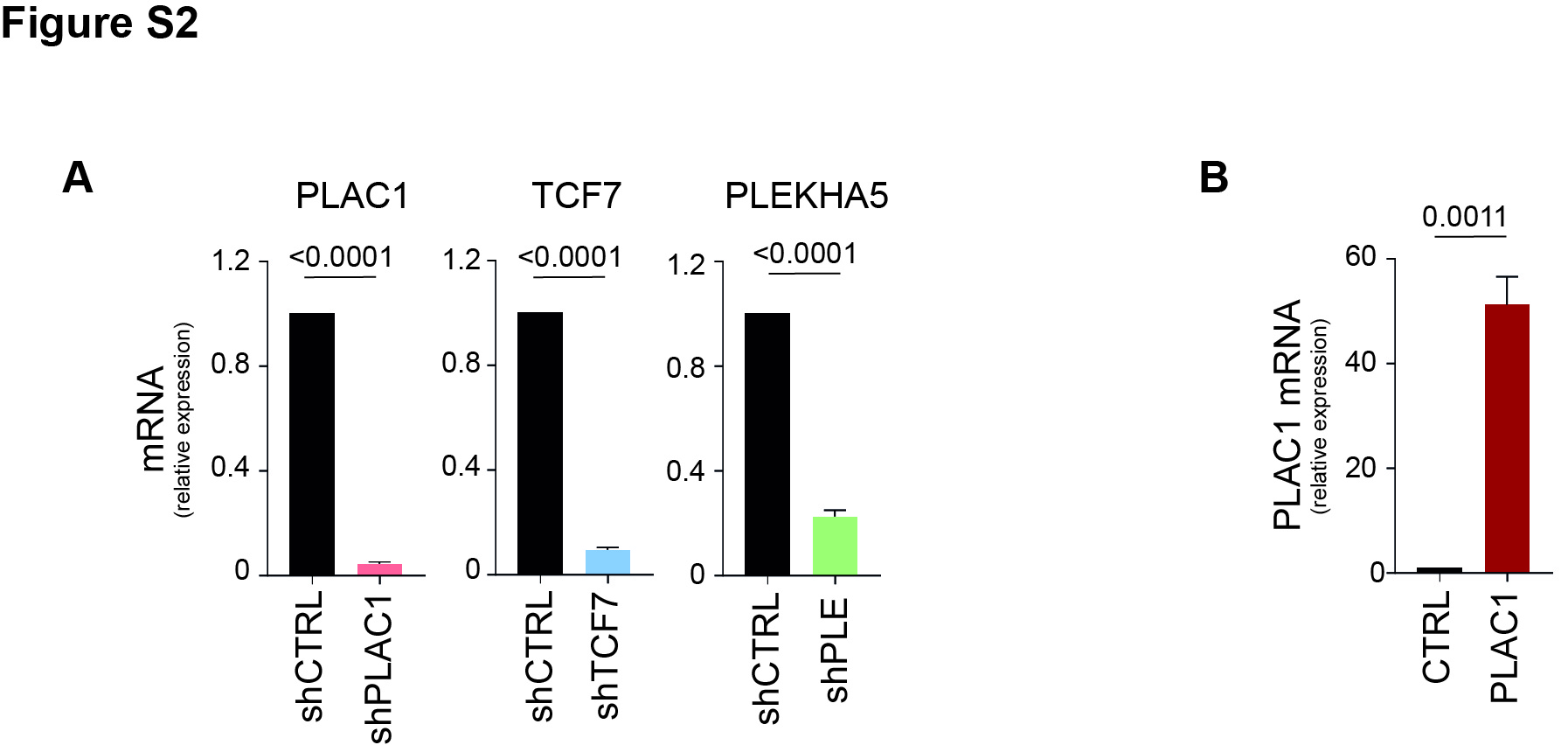

Supplement: Supplementary Figure 2 — Related to Figure 2 . PLAC1 regulates the activity of NK cells. (A) qPCR of PLAC1, TCF7 and PLEKHA5 expression in K562 cells transduced with lentiviral vectors encoding either control shRNA (shCTRL) or shRNA targeting PLAC1, TCF7 or PLEKHA5 genes (two-tailed unpaired Student t test). (B) qPCR of PLAC1 expression in K562 cells overexpressing PLAC1 (two-tailed unpaired Student t test). [file Image2.jpg]

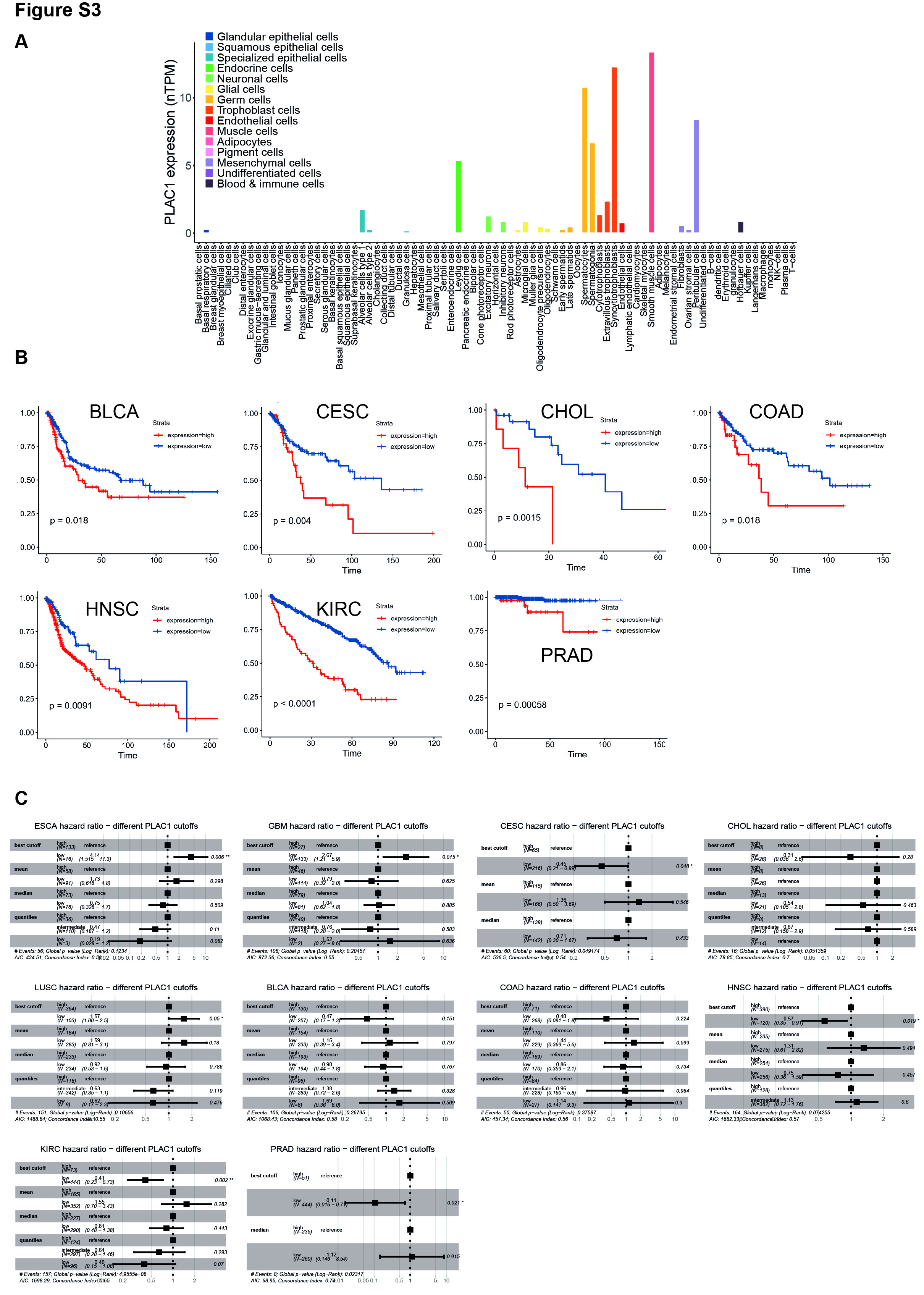

Supplement: Supplementary Figure 3 — Related to Figure 3 PLAC1 expression has prognostic value in tumors. (A) PLAC1 gene expression in the indicated human cell types. (B) Kaplan-Meier curves show the duration of overall survival of the indicated tumor patients according to the PLAC1 gene expression. (C) Forest plots showing hazard ratio and 95% confident intervals of the full range of cutoff values of the indicated tumor patients according to the PLAC1 gene expression. Log-rank test with Miller and Siegmund P-value correction was used. Statistically significant P values are indicated. [file Image3.jpg]

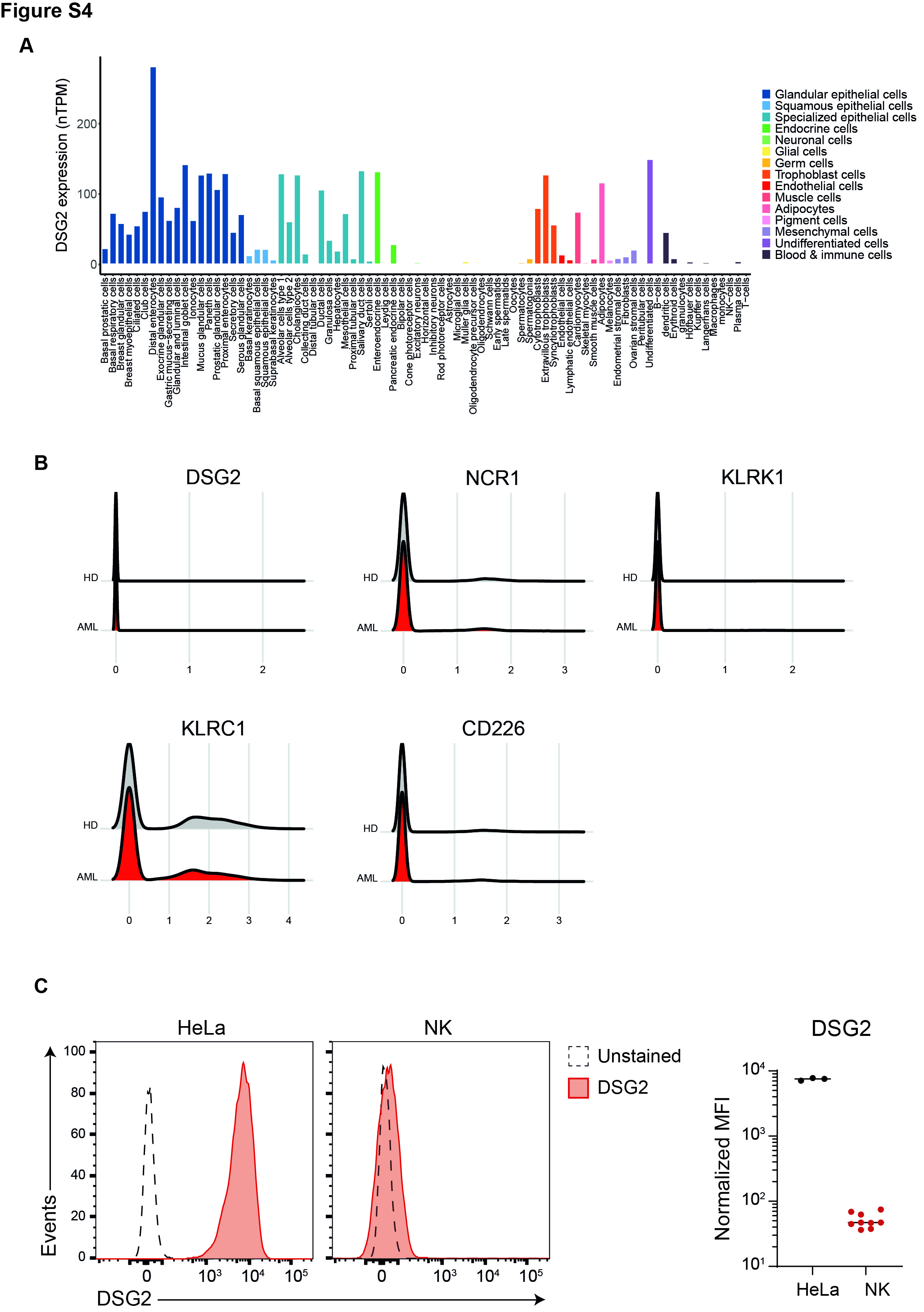

Supplement: Supplementary Figure 4 — Desmoglein 2 expression in tumor cells and NK cells. (A) DSG2 expression in the indicated human cells. (B) Density plots of DSG2 expression of NK cells from scRNA-seq of HD and AML patients (GSE159624). (C) Representative flow-cytometry analyses of DSG2 expression in HeLa cells and NK cells from a HD. Summary of DSG2 expression in NK cells from 3 HDs is reported on the right. [file Image4.jpg]
