# Supplementary material for: A genome-wide shRNA screen uncovers a novel potential ligand for NK cell activating receptors
Source: Front Immunol. 2025 Jun 18;16:1537876. doi: 10.3389/fimmu.2025.1537876 (PMC12213676; doi:10.3389/fimmu.2025.1537876)
Supplement: Supplementary file 5 [file Table1.docx]

**Table 1**

**List of genes identified in K562 cells resistant to NK cell lysis after single cell cloning**

| **Gene symbol** | **ID** | **GENE TARGET** |
| --- | --- | --- |
| ZBTB41 | NM_194314 | Zinc finger and BTB domain containing 41 |
| CYP2C19 | NM_124020 | Cytochrome P450, family 2, subfamily C, polyp.19 |
| TICAM2 | NM_021649 | Toll-like receptor adaptor molecule 2 |
| NUMB | NM_003744 | Numb homolog |
| PCSK2 | NM_002594 | Proprotein convertase subtilisin/kexin type 2 |
| PRAME | NM_206953 | Preferentially expressed antigen in melanoma |
| LOC390975 | NM_372749 | Similar to SH3-binding kinase |
| PLAC1 | NM_021796 | Placenta-specific 1-like |
| TCF7 | NM_003202 | Transcription factor 7 |
| WBSCR22 | NM_017528 | Williams Beuren syndrome chromosome region 22 |
| SRD5A2 | NM_000348 | Steroid-5-alpha-reductase, alpha polypeptide 2 |
| CD38 | NM_001775 | CD38 molecule |
| SSR4 | NM_006280 | signal sequence receptor, delta |
| DNAJB14 | NM_024920 | DnaJ (Hsp40) homolog, subfamily B, member 14 |
| PLEKHA5 | NM_019012 | Pleckstrin homology, family A member 5 |
