# Supplementary material for: A genome-wide shRNA screen uncovers a novel potential ligand for NK cell activating receptors
Source: Front Immunol. 2025 Jun 18;16:1537876. doi: 10.3389/fimmu.2025.1537876 (PMC12213676; doi:10.3389/fimmu.2025.1537876)
Supplement: Supplementary file 6 [file Table2.docx]

**Table 2**

**PLAC1 expression in TCGA tumors and normal tissues**

| **Cancer** | **Abbreviation** | **Group** | **mean** | **sd** |
| --- | --- | --- | --- | --- |
| Adrenocortical carcinoma | ACC | Tumor | 1.05 | 1.71 |
| Bladder Urothelial Carcinoma | BLCA | Normal | 0.88 | 0.91 |
| Bladder Urothelial Carcinoma | BLCA | Tumor | 1.51 | 1.33 |
| Breast invasive carcinoma | BRCA | Normal | 0.14 | 0.18 |
| Breast invasive carcinoma | BRCA | Tumor | 1.43 | 1.06 |
| Cervical squamous cell carcinoma and endocervical adenocarcinoma | CESC | Normal | 0.11 | 0.09 |
| Cervical squamous cell carcinoma and endocervical adenocarcinoma | CESC | Tumor | 1.48 | 1.28 |
| Cholangiocarcinoma | CHOL | Normal | 0.00 | 0.00 |
| Cholangiocarcinoma | CHOL | Tumor | 0.09 | 0.15 |
| Colon adenocarcinoma | COAD | Normal | 0.17 | 0.13 |
| Colon adenocarcinoma | COAD | Tumor | 0.70 | 0.77 |
| Lymphoid Neoplasm Diffuse Large B-cell Lymphoma | DLBC | Tumor | 0.29 | 0.54 |
| Esophageal carcinoma | ESCA | Normal | 0.37 | 0.67 |
| Esophageal carcinoma | ESCA | Tumor | 1.11 | 1.00 |
| Glioblastoma multiforme | GBM | Normal | 0.06 | 0.06 |
| Glioblastoma multiforme | GBM | Tumor | 0.49 | 0.65 |
| Head and Neck squamous cell carcinoma | HNSC | Normal | 0.17 | 0.22 |
| Head and Neck squamous cell carcinoma | HNSC | Tumor | 1.78 | 1.27 |
| Kidney Chromophobe | KICH | Normal | 0.06 | 0.06 |
| Kidney Chromophobe | KICH | Tumor | 0.11 | 0.28 |
| Kidney renal clear cell carcinoma | KIRC | Normal | 0.05 | 0.08 |
| Kidney renal clear cell carcinoma | KIRC | Tumor | 0.13 | 0.23 |
| Kidney renal papillary cell carcinoma | KIRP | Normal | 0.05 | 0.04 |
| Kidney renal papillary cell carcinoma | KIRP | Tumor | 0.10 | 0.35 |
| Acute Myeloid Leukemia | LAML | Tumor | 0.14 | 0.26 |
| Brain Lower Grade Glioma | LGG | Tumor | 0.29 | 0.24 |
| Liver hepatocellular carcinoma | LIHC | Normal | 0.05 | 0.08 |
| Liver hepatocellular carcinoma | LIHC | Tumor | 0.12 | 0.36 |
| Lung adenocarcinoma | LUAD | Normal | 0.07 | 0.08 |
| Lung adenocarcinoma | LUAD | Tumor | 0.94 | 0.98 |
| Lung squamous cell carcinoma | LUSC | Normal | 0.09 | 0.12 |
| Lung squamous cell carcinoma | LUSC | Tumor | 1.61 | 1.38 |
| Mesothelioma | MESO | Tumor | 0.46 | 0.81 |
| Ovarian serous cystadenocarcinoma | OV | Tumor | 1.04 | 1.31 |
| Pancreatic adenocarcinoma | PAAD | Normal | 0.16 | 0.10 |
| Pancreatic adenocarcinoma | PAAD | Tumor | 0.42 | 0.55 |
| Pheochromocytoma and Paraganglioma | PCPG | Normal | 0.02 | 0.03 |
| Pheochromocytoma and Paraganglioma | PCPG | Tumor | 0.07 | 0.09 |
| Prostate adenocarcinoma | PRAD | Normal | 0.05 | 0.12 |
| Prostate adenocarcinoma | PRAD | Tumor | 0.10 | 0.20 |
| Rectum adenocarcinoma | READ | Normal | 0.12 | 0.07 |
| Rectum adenocarcinoma | READ | Tumor | 0.86 | 0.86 |
| Sarcoma | SARC | Normal | 1.10 | 1.28 |
| Sarcoma | SARC | Tumor | 1.25 | 1.63 |
| Skin Cutaneous Melanoma | SKCM | Normal | 0.07 | N/A |
| Skin Cutaneous Melanoma | SKCM | Tumor | 0.54 | 0.72 |
| Stomach adenocarcinoma | STAD | Normal | 0.12 | 0.13 |
| Stomach adenocarcinoma | STAD | Tumor | 0.60 | 0.79 |
| Testicular Germ Cell Tumors | TGCT | Tumor | 1.51 | 1.17 |
| Thyroid carcinoma | THCA | Normal | 0.05 | 0.06 |
| Thyroid carcinoma | THCA | Tumor | 0.12 | 0.20 |
| Thymoma | THYM | Normal | 0.07 | 0.10 |
| Thymoma | THYM | Tumor | 0.13 | 0.32 |
| Uterine Corpus Endometrial Carcinoma | UCEC | Normal | 0.32 | 0.31 |
| Uterine Corpus Endometrial Carcinoma | UCEC | Tumor | 0.52 | 0.86 |
| Uterine Carcinosarcoma | UCS | Tumor | 2.48 | 1.68 |
| Uveal Melanoma | UVM | Tumor | 0.04 | 0.06 |

*N/A: Not applicable
